# The Hospital Officer's Bookshelf

**Published:** 1912-02-03

**Authors:** 


### THE HOSPITAL OFFICER'S BOOKSHELF.

Album of Photographs. Issued by the South Indian Branch of the British Medical Association. (London: John Bale, Sons and Danielsson. 1911. Price 10s. 6d. net.)

The vast amount of pathological material which exists in India deserves to be brought under the light of competent classification and investigation, and this Album will be welcomed as a first step in the right direction. As a collection of photographs it is a most successful production; as an instructive record the Album is of the greatest value. It is to be hoped that the efforts of those officers of the Indian Medical Service who have made so propitious a start will be seconded by a sustained and continuous endeavour on the part of the district medical officers all over India to make available for the use of investigators those surgical and pathological curiosities which abound among such an enormous population as this. The Album has been published through the generosity of the South Indian Branch of the British Medical Association, and is a most creditable collection of interesting photographs elegantly reproduced.

The Prevention of Dental Caries. By J. Sim Wallace, D.Sc., M.D., L.D.S. (London: The Office of the *Dental Record*. 1911. Price 1s. 6d.)

The modern teaching of dentists in regard to caries of the teeth and its relation to diet is but slowly being recognised. Text-books for medical students and practitioners do not realise as yet the full importance of the subject, but only good would result from requiring more attention to be paid by students to the principles of oral hygiene. It is true that these principles are perhaps not yet clearly consolidated, but the newer text-books are now laying stress on the greater importance of the kind of food taken rather than on antiseptic mouth-washes and tooth-brushes. Dr. Sim Wallace in this essay points out in a convincing manner the great benefits accruing from a diet of a kind to stimulate mastication, and considers it equally important that the last food taken should tend to leave the mouth clean, or at any rate free from carbohydrate. This probably is the reason that led to the first cup of coffee being taken after dinner, and also explains why it should be drunk then in all astringency without the sticky addition of milk. Thus he inveighs against the practice of rounding off a meal with bread and jam or a sweet pappy pudding. One of the best means of cleaning the mouth is the mastication of a raw apple, and the author says that children who habitually end their meal with fresh fruit are far less prone to indulge in sweetmeats. Many other aspects of the subject of dental caries are dealt with in Dr. Wallace's little book, and we can cordially direct the attention of medical men as well as dentists to the suggestive and profitable matter contained therein.

School Life. (National Health Manuals, No. 3.) Edited by T. N. Kelynack, M.D. (London: Chas. Kelly. 1911. Pp. 160. Price 1s. net, limp cloth; 1s. 6d. net, cloth boards.)

The ambitious title and aims of this small volume indicate that it is intended to fill a gap in popular serious literature which unquestionably exists. The list of contents still further shows that the system on which the work is arranged has also been carefully thought out; for there are twelve chapters, each by a different author, dealing with every aspect of the economies and of the life of the school child. Unfortunately the result cannot honestly be called satisfactory, or anything like it. The book is simply a collection of scrappy short essays, each containing a few platitudes, and none of them of any real value to the hygienist, the parent, or the teacher. The best is that on dental condition in school children, by Mr. C. E. Wallis. But, on the whole, the volume is distinctly disappointing; it leaves one with the impression of a catchpenny production which merely tinkers with an important subject well worthy of a better treatment.

Conduct and its Disorders, Biologically Considered. By Charles A. Mercier, M.D., F.R.C.P., F.R.C.S. (London: Macmillan and Co., Ltd. 1911.

Written in his trenchant and forcible style, this book is mainly Dr. Mercier's exposition of his views on conduct. It speaks much for the author's ability and his successful handling of a difficult and hackneyed subject that he has been able to carry the reader's interest with him through nearly four hundred closely printed pages. In these he deals exhaustively, yet always lucidly and in a common-sense way which appeals strongly to the reader's "logical sense," with the manifold aspects of conduct in relation to morality, with various types of conduct, and with their influences upon the individual and his environment. To his professional colleagues Dr. Mercier's views on the subject are more or less well known; nevertheless we welcome this clear exposition, especially as the author has treated certain aspects of the subject, which are usually written and spoken of in a very guarded fashion, in a way that compels attention. Such a book ought to be popular in the best sense. It ought to appeal—and it does appeal—to every practitioner of medicine, and, moreover, to that large section of the public that can discuss the subjects with which it deals in a sane and temperate fashion. The reading of some of the chapters ought, for instance, to dispel the loose sentimentality with which several aspects of very important social and ethical problems are yet veiled. That some of these chapters are highly controversial goes without saying, but just in that fact, perhaps, lies the educative value of the book—in its possibility, namely, of arousing knowledgable criticism. We congratulate Dr. Mercier on his work, which will go far to enhance his reputation as one of our foremost and most original writers on ethical questions.

An Index of Treatment. By Various Writers. Edited by Robert Hutchison, M.D., F.R.C.P., and H. Stansfield Collier, F.R.C.S. (Bristol: John Wright and Sons, Ltd. 1911. Price 21s. net.)

This is the sixth edition of a text-book which is already deservedly popular with both practitioners and students. It has been revised and brought up to date, and is as admirable a compilation as one can wish for. Several new articles are included, among them being chapters on puerperal sepsis, photo and thermo therapy, treatment by solid carbonic acid gas, and several others. We notice no important omissions, but it is obvious that there must be extreme condensation in a manual that attempts to deal with the whole field of medicine and surgery within the limits of little more than a thousand pages. The alphabetical order which is followed may give rise to a little inconvenience, for it is not always easy to find the particular disease which one looks for. We turn, for example, to "Pancreas," but find that the diseases of this gland are not discussed under that heading, and it is a matter of considerable difficulty to find out what treatment the writers recommend in a case of acute hæmorrhagic pancreatitis. A few points seem to us to be open to discussion with regard to the treatment advised. In the note on flat-foot, written by so eminent an authority as Mr. Robert Jones, it is stated that the use "of arches inside the boot is fundamentally wrong"; we agree, if the proviso is added that ordinary "braces" are meant, but the experience of Continental orthopædists is distinctly in favour of using a properly fitted celluloid inset in such cases. Many new remedies and modes of treatment are conspicuous by their absence. In a text-book this is perhaps desirable, since the multitude of new remedies is so large that the practitioner is often in a quandary which to use, especially where the indications are not laid down with precision. There is a certain amount of equally necessary dogmatism in all the articles which makes the work very useful to the student who desires to revise his hospital notes. Taken as a whole the volume can be thoroughly recommended. It should find a place on the shelves of every practitioner, and the newly qualified man who is starting practice cannot do better than invest a guinea in buying it.

Manual of Surgery. By Alexis Thomson, F.R.C.S. Edin., and Alexander Miles, F.R.C.S. Edin. (Edinburgh, Glasgow, and London: Henry Frowde and Hodder and Stoughton. 1911. Price 10s. 6d. net.)

This is the fourth edition of the first volume of what is familiarly known to most of us as "Thomson and Miles." We notice that the authors have considerably extended the scope of this very popular text-book. Formerly in two volumes only, the fourth edition will be in three, the third volume being devoted to operative surgery, while the second deals with regional surgery. The first volume, which we have before us, deals with general surgery. In reviewing the former editions we have stated that the work is one of the best of its kind in the English language. In reviewing the present edition we can thoroughly reiterate that verdict. Every chapter is admirably written, containing the maximum of information compressed into the minimum of space, and so compressed withal that it is not a task to read it. If we may be allowed a special criticism with regard to the illustrations, we would point out that the blocks on pages 48 and 567 ought to be altered. The one shows a bad way of applying a Biers' bandage (which should be applied as far away from the joint as possible), and the other an inaccurate way of applying a Hodgens splint. The majority of the new illustrations are admirable. As a book for the student—and, be it added, for the practitioner who has not the time to read larger manuals, but desires a handy compendium to which he can refer as occasion arises—the book is one of the most suitable which we have read, and its convenient size and careful arrangement of subjects will appeal to those who wish to buy a new handbook of surgery.

Dolls, Dead and Alive. By Otto Ernst. Translated by A. C. Caton, 22 Mount Carmel Chambers, Kensington. (London: A. C. Caton. 1911. 1s. 6d. net.)

To Otto Ernst belongs the credit of having initiated that vigorous protest against the canting hypocrisy in art which some people innocently style modern realism in literature. When one turns from so unpleasant and hopelessly inartistic and crude a representation of fact as Mr. Barker's "Waste" to Ernst's first and consequently immature attempt "Jugend," one realises to the full the difference between creative art and the literary kodakking of the muck-heap. To English readers Ernst is unfortunately not so well known as he ought to be, and the translator of "Dolls" has done well to present him in such clear and simple language. "Dolls" is essentially a book for parents. It deals with psychological questions, not in the coldly analytical manner of Freud, but in a poetical and altogether charming fashion which makes it a delight to read. Here and there we think the translator has missed the tense vigour of the original, but on the whole the task has been excellently done, and the book deserves the attention of every father or mother whose children possess dolls and are young enough to take an interest in Hans Andersen.

Nerves and the Nervous. By Edwin Ash, M.D. Lond. (London: Mills and Boon, Ltd. 1911. Pp. 239. Price 5s. net.)

A book written with a view to the instruction of the lay reader on the subject of "nerves" will doubtless at the present day receive a fair share of popularity. Dr. Edwin Ash appears to have carried out this intention with more than average success. A considerable amount of very beneficent advice is to be found among its pages, and to those who prefer to consult a book rather than a physician this volume will prove distinctly helpful if they act on the excellent admonitions contained therein. The chapter on "Psychic Healing" strikes one as being unnecessary, to say the least; but such chapters as those on self-help for nervous people, on diet, alcohol, etc., and those earlier ones explaining what is meant by "nerves," fulfil their object very well. Again, the upbringing of nervous children is always a matter of great importance, and in the last chapter the author has presented the chief points to be attended to by those who have to educate boys and girls of nervous temperament. The directions given as to such important factors as diet, baths, sleep, rest, and so on, can readily be made applicable to individuals; but the author wisely warns his public that they should in no wise allow these directions to replace expert advice. On the whole, this book may be of practical value to a great many people who are worried either by their own nerves or by the nerve troubles of their acquaintances.

Child Nurture. By Honnor Morten. (London: Mills and Boon, Ltd. 1911. Pp. 243. Price 3s. 6d. net.)

In later days it is quite possible that aviation, wireless telegraphy, and similar scientific marvels will be less regarded as the peculiar glory of the early twentieth century than the increasing and awaking sense of national and individual responsibility for the well-being of the child. Child-study societies and propaganda, child-study magazines, pamphlets, articles, and books spring up on all sides; and though the intent is mostly good, some of this miscellaneous literature is not of a very high order. The latest addition to it is the volume on child nurture by Miss Morten. It is somewhat ambitious, and great pains have evidently been bestowed on it. With much of the contents a most cordial agreement is quite easy; and much of the advice given is thoroughly sound. Yet candour compels one to admit that the author has undertaken a task which is in many respects beyond her powers. In particular she should have got some competent medical authority to read her proofs, for she would thus have been saved from many ridiculous assertions; the chapter on heredity and environment teems with the gravest errors, each enunciated with the utmost assurance as if it were a well-ascertained fact. The fourth chapter would have been far better omitted altogether, especially as the lessons it may appear to some people to be inculcating are bottle-feeding and the avoidance of vaccination. The elaborate dissection of child psychology is also somewhat unconvincing; the author insists too much upon the abnormal child and its peculiarities. We applaud her intentions and her motives, but feel compelled to speak with greater reserve of her performance.

